# NGF contributes to activities of acid‐sensing ion channels in dorsal root ganglion neurons of male rats with experimental peripheral artery disease

**DOI:** 10.14814/phy2.15933

**Published:** 2024-02-05

**Authors:** Qin Li, Jianhua Li

**Affiliations:** ^1^ Heart and Vascular Institute The Pennsylvania State University College of Medicine Hershey Pennsylvania USA

**Keywords:** acid‐sensing ion channels, limb ischemia and reperfusion, muscle afferent nerve, peripheral artery disease

## Abstract

A feature of peripheral artery diseases (PAD) includes limb ischemia/reperfusion (I/R) and ischemia. Both I/R and ischemia amplify muscle afferent nerve‐activated reflex sympathetic nervous and blood pressure responses (termed as exercise pressor reflex). Nevertheless, the underlying mechanisms responsible for the exaggerated autonomic responses in PAD are undetermined. Previous studies suggest that acid‐sensing ion channels (ASICs) in muscle dorsal root ganglion (DRG) play a leading role in regulating the exercise pressor reflex in PAD. Thus, we determined if signaling pathways of nerve growth factor (NGF) contribute to the activities of ASICs in muscle DRG neurons of PAD. In particular, we examined ASIC1a and ASIC3 currents in isolectin B_4_‐negative muscle DRG neurons, a distinct subpopulation depending on NGF for survival. Hindlimb I/R and ischemia were obtained in male rats. In results, femoral artery occlusion increased the levels of NGF and NGF‐stimulated TrkA receptor in DRGs, whereas they led to upregulation of ASIC3 but not ASIC1a. In addition, application of NGF onto DRG neurons increased the density of ASIC3 currents and the effect of NGF was significantly attenuated by TrkA antagonist GW441756. Moreover, the enhancing effect of NGF on the density of ASIC3‐like currents was decreased by the respective inhibition of intracellular signaling pathways, namely JNK and NF‐κB, by antagonists SP600125 and PDTC. Our results suggest contribution of NGF to the activities of ASIC3 currents via JNK and NF‐κB signaling pathways in association with the exercise pressor reflex in experimental PAD.

## 1 INTRODUCTION

Exercise activity elevates sympathetic nerve activity (SNA) and blood pressure (BP), due to “Exercise Pressor Reflex” (EPR) and “Central Command” (Coote et al., 1971; Mitchell et al., 1983; Waldrop et al., 1996). The EPR is a neural mechanism by which metabolic and mechanical receptors of muscle afferents (Groups III and IV) are stimulated during muscle contraction and then activate the CNS (Kaufman, 1996). (Kaufman, 1996; Mitchell et al., 1983). The central command is a corresponding activation of motor and sympathetic systems thus causing increased autonomic activities during exercise. Nonetheless, exercise‐evoked amplification of EPR in peripheral artery disease (PAD) needs to be studied (Baccelli et al., 1999; Bakke et al., 2007; Ritti‐Dias et al., 2011).

Approximately 200 million PAD patients are diagnosed worldwide (Criqui & Aboyans, 2015; Fowkes et al., 2017), who are at a high risk of various cardiovascular disorders such as myocardial infarctions and cerebral vascular accidents because a higher BP response is seen during exercise (Anand et al., 2018; Bauersachs et al., 2019; Ouriel, 2001). An SNA‐driven BP exaggeration also contributes to poor clinical outcomes (Piepoli et al., 1999; Ponikowski et al., 2001) linking to a lower survival of PAD patients (De II et al., 2008). Also, “intermittent claudication,” caused by limb ischemia during walking in PAD patients is a main issue affecting patients' daily activities (Hart et al., 2018). Thus, in order to provide clinical evidence for human studies, additional investigations of the underlying signaling pathways responsible for the abnormality in the EPR in PAD are demanding.

Numerous previous reports using animal models for studying human PAD suggest that some neural pathways and receptors are engaged in the exaggerated EPR, including oxidative stress and proinflammation, and acid‐sensing ion channels (ASICs), purinergic P2Xs and bradykinin receptors, etc. (Liu et al., 2010; Stone & Kaufman, 2015; Teixeira & Vianna, 2022; Xing et al., 2012). Worth mentioning, some of those receptor mechanisms were observed in human studies (Campos et al., 2019; Muller et al., 2012).

Numerous metabolites are observed in exercising skeletal muscles, namely lactic acid, accompanied with a lower pH level, and they stimulate ASICs on the free endings of the thin fiber afferent nerves (Kaufman & Hayes, 2002; Sinoway & Li, 2005). Lactate, acidic phosphate, and H^+^ are the endogenous substances to activate ASICs in evoking and/or modulating the EPR (Campos et al., 2019; Hayes et al., 2007, 2008; Immke & McCleskey, 2001; Liu et al., 2010; Osmakov et al., 2014; Stone et al., 2015; Stone & Kaufman, 2015; Tsuchimochi et al., 2011). Using a rat model of PAD, ASIC1a and ASIC3 have been reported to contribute to the EPR and ASIC3 has a major role in the exaggerated reflex activity (Farrag et al., 2017; Kim et al., 2020; Xing et al., 2012).

Employing a surgical procedure of femoral artery occlusion (FAO) numerous investigations have been conducted to examine the EPR (Kim et al., 2020; Li et al., 2020, 2021a; Liu et al., 2010; Stone et al., 2015; Tsuchimochi et al., 2011; Xing et al., 2012). FAO imitates a disorder of insufficient blood flow to the contracting limb muscles during exercise in PAD patients (Waters et al., 2004). However, in PAD patients, in addition to the ischemia stage, a recovery of the blood supply to the limb muscles during resting stage leads to a pathophysiological condition, known as ischemia/reperfusion (I/R) injury (Vun et al., 2016). Thus, ischemia may not totally reflect pathophysiology of the exercising muscles in PAD patients. Additionally, the mechanisms responsible for limb I/R injury and ischemia are distinct in PAD (Hamburg & Creager, 2017; Simon et al., 2018), which may differently affect the EPR in animals with hindlimb I/R and in animals with FAO. Therefore, it is important to determine the EPR under both conditions in PAD.

On the basis of the neurochemical and neurotrophic characteristics, there are two distinct neuronal groups of dorsal root ganglion (DRG), namely isolectin B_4_‐positive and ‐negative (IB4+ and IB4−) (Averill et al., 1995; Bennett et al., 1998; Michael et al., 1997; Molliver et al., 1997). IB4+ neurons are relatively “peptide poor,” but express a surface carbohydrate binding IB_4_ and these neurons contain receptors of glial cell line‐derived neurotrophic factor (GDNF). On the other hand, IB4− DRG neurons containing trkA receptors responsive to nerve growth factor (NGF) depend on NGF for survival. In these neurons, calcitonin gene‐related peptide and substance P are identified. Our recent study suggests that the hindlimb I/R and FAO affect ASIC signaling pathways in IB4− DRG neurons (Li et al., 2023). The role played by NGF in regulating the activities of ASIC currents is also likely via JNK and NF‐κB signaling pathways (Mamet et al., 2003; Matricon et al., 2013; Wei et al., 2020).

Accordingly, we examined the protein levels of NGF and its receptors, and ASIC1a and ASIC3 in the L4‐6 DRGs innervating the hindlimb muscle of I/R rats and FAO rats. We then examined the effects of NGF on ASIC1a and ASIC3 currents in rat IB4− L4‐6 DRG neurons, and signaling pathways JNK and NF‐κB in engagement of NGF regulating the activities of ASIC1a and ASIC3 currents. We hypothesized that NGF plays an essential role in regulating the activities of ASIC currents in the DRG neurons likely via JNK and NF‐κB signaling leading to the exaggerated EPR in PAD.

## 2 MATERIALS AND METHODS

### 2.1 Ethical approval

All experimental procedures were approved by the Institutional Animal Care and Use Committee of Penn State College of Medicine (Protocol#: PRAMS201147671) and were conducted in accordance with the National Institutes of Health Guide for the Care and Use of Laboratory Animals. A total of 76 male Sprague–Dawley rats (4–6 weeks old; body weight ranging from 150 to 200 g) were purchased from Charles River Laboratories and housed in accredited temperature and ventilation‐controlled facilities with a 12:12 h light–dark cycle and ad libitum access to standard rat chow and water. Note that only male animals were included in the current study because our main study focus was to examine the ASIC currents in vitro methods and sex discrepancies should be considered in a study with the whole animal preparations.

### 2.2 Experimental models of hindlimb ischemia and I/R

The rats were anesthetized by inhalation of an isoflurane‐oxygen mixture (2%–5% isoflurane in 100% oxygen). As described previously (Li et al., 2020, 2021a), to obtain FAO rats the femoral artery on the hindlimbs was surgically exposed, dissected, and ligated ~3 mm distal to the inguinal ligament. The incision and skin were then closed with surgical staples. To obtain hindlimb I/R group, the ligation procedures were performed and then the incision and skin were closed with surgical staples. The rats were kept in normal air of the surgery room without anesthesia for 6 h and then by inhalation of an isoflurane–oxygen mixture the ligation of femora artery was reopened to recover the blood flow into the femoral artery and the surgical incision and skin were closed. The same procedures were performed on the contralateral hindlimbs with except that a suture was placed below the femoral artery, but the artery was not tied for the western blotting study and the same sham procedures were performed in a different group of rats for the ELISA and patch clamp study. After the surgery, the FAO rats were returned to the cage for 24‐h regular housing and I/R rats for 18 h before the designed experiments were conducted.

In order to relieve postoperative pain of rats, buprenorphine hydrochloride (0.05 mg/kg, subcutaneously) was given prior to the surgery and the rats were kept in the surgery room for 2–3 h for careful observation after the surgery, and then returned to the animal facility.

### 2.3 Enzyme‐linked immunosorbent assay

Rats were anesthetized with isoflurane and decapitated, and then L4‐L6 DRG tissues in both sides were exposed, removed, and dissected. Both ipsilateral and contralateral sides of the L4‐L6 DRGs from the same rat were removed 24 h after the end of femoral artery ligation and 18 h after the end of I/R procedures (I/R group). β‐NGF levels (Bax et al., 1997) in the rat L4‐L6 DRGs were assayed with a modified ELISA (Li et al., 2021b). This assay was performed at room temperature as instructed by the manufacturer (RayBiotech Co. Cat# ELR‐bNGF‐CL). Briefly, the diluted samples (2 mg/mL) and rat β‐NGF standard solution were distributed in polystyrene 96‐well immunoplates (100 μL per well; Thermo‐Fisher Co. Cat#9205) and incubated for 2.5 h with gentle shaking. After being thoroughly washed for four times, 100 μL of diluted biotin‐labeled antibody mixture was added to each well and incubated for 1 h. After thoroughly washing, 100 μL of diluted streptavidin‐HRP conjugate (Invitrogen Co. Cat#43423) per well was added and incubated for 45 min. Then 100 μL of substrate per well was incubated for 30 min in the dark, then 50 μL of stop solution was added. The optical density at 450 nm was immediately examined using an ELISA reader (BioTek).

### 2.4 Western blotting analysis

Rats were anesthetized with isoflurane and decapitated, and then L4‐L6 DRG tissues in both sides were exposed, removed, and dissected. Note that both ipsilateral and contralateral sides of the L4‐L6 DRGs from the same rat were removed 24 h for FAO group and 18 h for I/R group. As described previously (Li et al., 2021a), the western blotting procedures and analysis were performed. In brief, a total protein of L4‐L6 DRGs from the ligated limbs/I/R limbs and their respective contralateral control limbs was extracted with RIPA buffer containing proteinase inhibitor cocktail (Sigma‐Aldrich Co. Cat# P8340) and quantified with BCA method (Thermo‐Fisher Sci. Cat# 23225). 20 μg of total protein was loaded in 10% Mini‐Protean TGX Precast gels (Bio‐Rad Co. Cat#4561034) after being boiled at 95°C for 5 min in SDS sample buffer, then electrophoretically transferred to polyvinylidene difluoride membrane (Thermo‐Fisher Sci. Cat# 88518; 0.45‐μm pore size). After blocking with 5% non‐fat milk in 0.1% Tween–TBS buffer for 1 h, the membrane was respectively incubated with rabbit anti‐ASIC1a (1:500, Alomone Lab Cat #ASC‐014), anti‐ASIC3 (1:500, Alomone Lab #CatASC‐018), anti‐p75NTR (1:1000, Cell Signaling Tech. Cat# 8238), and goat anti‐TrkA (1:2000, R&D Inc. Cat# AF1056) primary antibodies at 4°C overnight. Then they were incubated with HRP‐conjugated goat anti‐rabbit (1:3000, Abcam Co. Cat#ab6721) or donkey anti‐goat secondary antibody (1:3000, Thermo‐Fisher Co. Cat#A15999) at room temperature for 1 h. The immunoreactivity was visualized using an enhanced chemiluminescence system (Cell Signaling Tech Cat#6883). The membrane was stripped and incubated with mouse anti‐GAPDH primary antibody (1:4000, Thermo‐Fisher Co. Cat#MA5‐15738‐HRP) as an equal loading control of protein expression. The optical densities of targeted bands were analyzed using the NIH Image J Software.

### 2.5 Electrophysiology

#### 2.5.1 Labeling of hindlimb muscle afferent DRG neurons

As described previously (Li et al., 2020, 2021a), 2 days before I/R and FAO models were constructed, an incision in the calf area of the hindlimb was made and the gastrocnemius muscle was exposed after rats were anesthetized. The lipophilic dye 1, 1′‐dioctadecyl‐3, 3, 3′, 3′‐tetramethylindocarbocyanine perchlorate (DiI, 60 mg/mL, Thermo‐Fisher Co. Cat#E7510) was injected into the white portion of the gastrocnemius muscle. A total volume of 1 μL DiI tracer was administered at different locations, with the needle left in the muscle for 1 min to prevent the tracer leakage. Then, the rats were returned to their cages for the fluorescent DiI retrograde transported to DRGs to label muscle DRG neurons.

#### 2.5.2 Culture of DRG neurons

First, the rats were anesthetized with over 5% isoflurane and decapitated. Then, L4‐6 DRGs from both limbs were removed and dissected, and immediately transferred into ice‐cold Hank's balanced salt solution (HBSS, Sigma‐Aldrich Co. Cat#H1387). Following being freed from the connective tissues, the ganglia were digested and dissociated in Earle's balanced salt solution (Sigma‐Aldrich Co. Cat#E7510) containing collagenase Type D (0.6 mg/mL; Roche Inc Cat#70329522), trypsin (0.30 mg/mL; Worthington Biochem Co. Cat#9002), and DNase (0.1 mg/mL; Sigma‐Aldrich Co. Cat#D5025‐150KU), followed by shaking for 45 min at 34°C. The dissociated DRG neurons were seeded on 10% poly‐L‐lysine–coated coverslips (Dia^#^ 8 mm, Warner Co. Cat#64‐0701) in 35‐mm culture dish (Celltreat Sci. Cat#229638) containing 2 mL DMEM medium (Corning Co. Cat#10‐013‐CV) supplemented with 10% FBS (HyClone Lab. Cat#SH30109.03), 1% glutamine, and 1% penicillin–streptomycin. After this, the neurons were cultured at 37°C with 5% CO_2_, 95% air in a cell culture incubator (VWR Inc.).

#### 2.5.3 Recording ASIC currents in muscle DRG neurons

Modified from the previous studies (Liu et al., 2004; Poirot et al., 2006), the extracellular solutions: 140 mM NaCl, 5 mM KCl, 2 mM CaCl2, 1 mM MgCl2, 10 mM Mes (2‐(N‐morpholino) ethanesulfonic acid), 10 mM glucose, and pH was adjusted with 1 M NaOH. Holding at −70 mV, the seals (2–8 GΩ) were obtained using 3–5 MΩ resistance of glass electrodes which was filled with internal solution (in mM): 140 KCl, 2 MgCl2, 5 EGTA, 10 HEPES, 0.3 NaGTP, 2 MgATP, pH adjusted to 7.3 using 1 M KOH. A series of resistance was 70%–90% compensated to under 10 MΩ if necessary when the whole‐cell configuration was applied. In a voltage clamp mode, ASICs currents were recorded by using one gap‐free protocol of 30 s. We stored all the chemicals in the stock solutions, which were diluted in extracellular solution immediately before we used them. They were individually held in a series of independent syringes of the pressurized VC3‐8MP perfusion system (ALA Inc.). A distance of 100 μm was kept from the outlet tip mouth to examine the neuron and the recording chamber were continuously bathed in extracellular solution of pH 7.4.

ASICs currents of L4‐6 DRG neurons with diameters ≤35 μM were recorded in the whole‐cell configuration using a MultiClamp 700B amplifier supplied with Digitizer 1440A (Axon Inc). With application of pH 6.7 solution, each of patch clamp recordings was obtained from one individual DRG neuron of I/R and FAO rats. Signals were acquired by using pClamp10.1 and analyzed with pClampfit 10.7 software. If acid solution elicited an inward current of >50 pA in peak amplitude, we considered neurons as proton‐sensitive ones for analysis.

We conducted all patch clamp recordings on the DRG neurons within 24 h after their being dissociated. DRG neurons were identified as Dil‐positive (red color) under an inverted microscope with a fluorescent filter, and images were displayed on a video monitor. Meanwhile, immediately before patch clamp recording, IB4+ muscle neurons were identified by 5‐min incubation of IB_4_‐Alexa Fluor 488 (3 μg/mL in external solution, Invitrogen Co. Cat #I21411) (as green color) using a combination of fluorescence illumination and differential interference contrast (DIC; ×20–40) optics on a Nikon TE2000 inverted microscope. A subpopulation of IB4− L4‐6 DRG neurons were used for current recording and analysis. In this study, IB4− neurons were used because they have physiological features to study the regulatory effects of NGF on the activities of ASIC currents (Averill et al., 1995; Bennett et al., 1998; Michael et al., 1997; Molliver et al., 1997).

### 2.6 Statistical analysis

All data in this study were presented as mean ± SD (standard deviation). One‐way ANOVA was used to analyze expression of β‐NGF, and unpaired *t*‐test was used for expression of NGF receptors and ASIC receptors in DRGs. Two‐way or three‐way chi‐squared test (*χ* ^2^ test) was used to analyze the distribution of the neurons with ASICs currents. One‐way or two‐way ANOVA was applied to analyze the density of ASIC1a‐like and ASIC3‐like currents, and post hoc analysis with Tukey's tests were applied to compare the difference between specific groups. All statistical analyses were performed using SPSS v26 and the significant differences were considered at *p* < 0.05.

## 3 RESULTS

### 3.1 Expression of NGF and its receptors in DRGs

**FIGURE 1:**
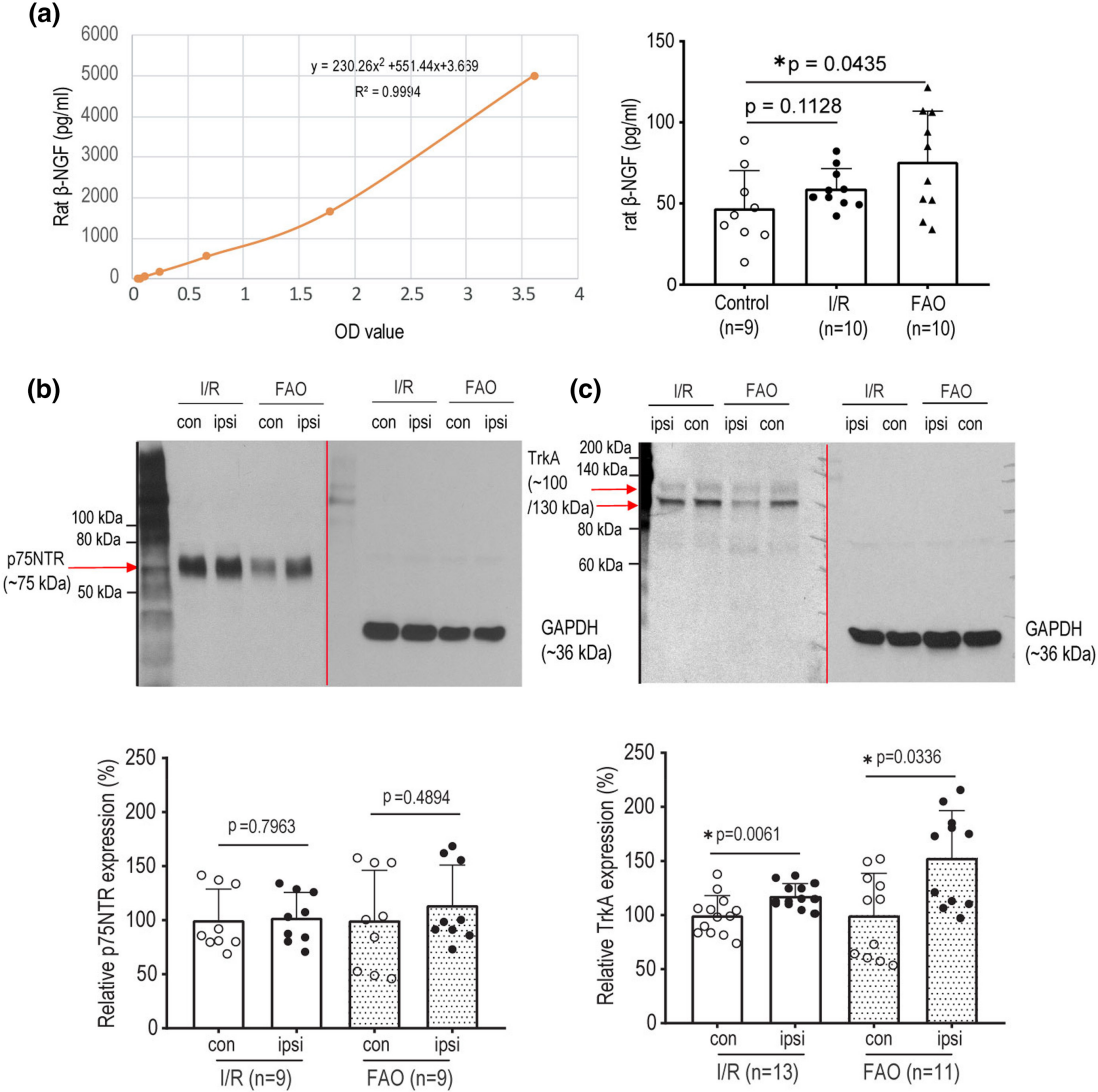
The levels of NGF and expression of p75NTR and TrkA receptors in rat L4‐6 DRGs. (a) Rat β‐NGF levels were analyzed using ELISA methods. The left panel is the standard curve for examination of rat β‐NGF, and the right panel is the graph of the averaged levels of rat β‐NGF level after hindlimb ischemia/reperfusion (I/R) and femoral artery occlusion (FAO). One‐way ANOVA was used to analyze the data (*F* = 4.354 and *p* = 0.0238 for main effect). *p =* 0.1128, comparing I/R (*n* = 10) and control (*n* = 9); and *p = 0.0435, comparing FAO (*n* = 10) and control groups (*n* = 9). Control group was obtained from DRG tissues of a separate group of rats. (b and c) The protein levels of p75NTR and TrkA expression were determined. The upper panels are respectively the representative western blotting bands of p75NTR and TrkA expression (vertical lines used to separate the panels of p75NTR/TrkA and GADPH); and the bottom panels are the graphs of the relative expression levels of p75NTR and TrkA. I/R and FAO did not significantly change p75NTR expression in DRGs. Un‐paired *t*‐test was used to analyze the data. *p* = 0.7963 between I/R and its control (*n* = 9 for each group); and *p* = 0.4894 between FAO and its control (*n* = 9 for each group). However, I/R and FAO significantly increased TrkA expression in DRGs. *p < 0.05, I/R (*n* = 13) and FAO (*n* = 11) versus their respective contralateral controls. That is, *p* = 0.0061 between I/R and its control (*n* = 13); and *p* = 0.0336 between FAO and its control (*n* = 11). Both ipsilateral and contralateral sides of the L4‐L6 DRGs from the same rat were removed for I/R and FAO groups. “ipsi” designates the limbs treated with I/R or FAO and “con” designates the contralateral limbs with the sham procedures as respective controls. Individual data are also shown in figures. The membrane was stripped and incubated with mouse anti‐GAPDH primary antibody as an equal load control.

We first determined the levels of rat β‐NGF in L4‐6 DRGs, and expression of p75NTR and TrkA receptors. The new findings are presented in Figure 1. That is, Figure 1a shows that FAO (*n* = 10) significantly amplified the levels of β‐NGF in DRGs as compared with the control group (*p =* 0.0435 vs. control/*n* = 9), but a significant increase in β‐NGF was not observed after I/R (*n* = 10; *p =* 0.1128 vs. control/*n* = 9). In addition, Figure 1b,c (representative bands and averaged data) demonstrate that p75NTR expression was not significantly altered in DRGs of I/R limbs and FAO limbs (*p* = 0.7963 between I/R/*n* = 9 and its control/*n* = 9; and *p* = 0.4894 between FAO/*n* = 9 and its control/*n* = 9). However, TrkA expression in DRGs was significantly amplified in the I/R and FAO groups compared with their respective contralateral controls (*p* = 0.0061, I/R/*n* = 13 vs. its control/*n* = 13; and *p* = 0.0336, FAO/*n* = 11 vs. its control/*n* = 11).

### 3.2 Expression of ASIC1a and ASIC3 in DRGs

**FIGURE 2:**
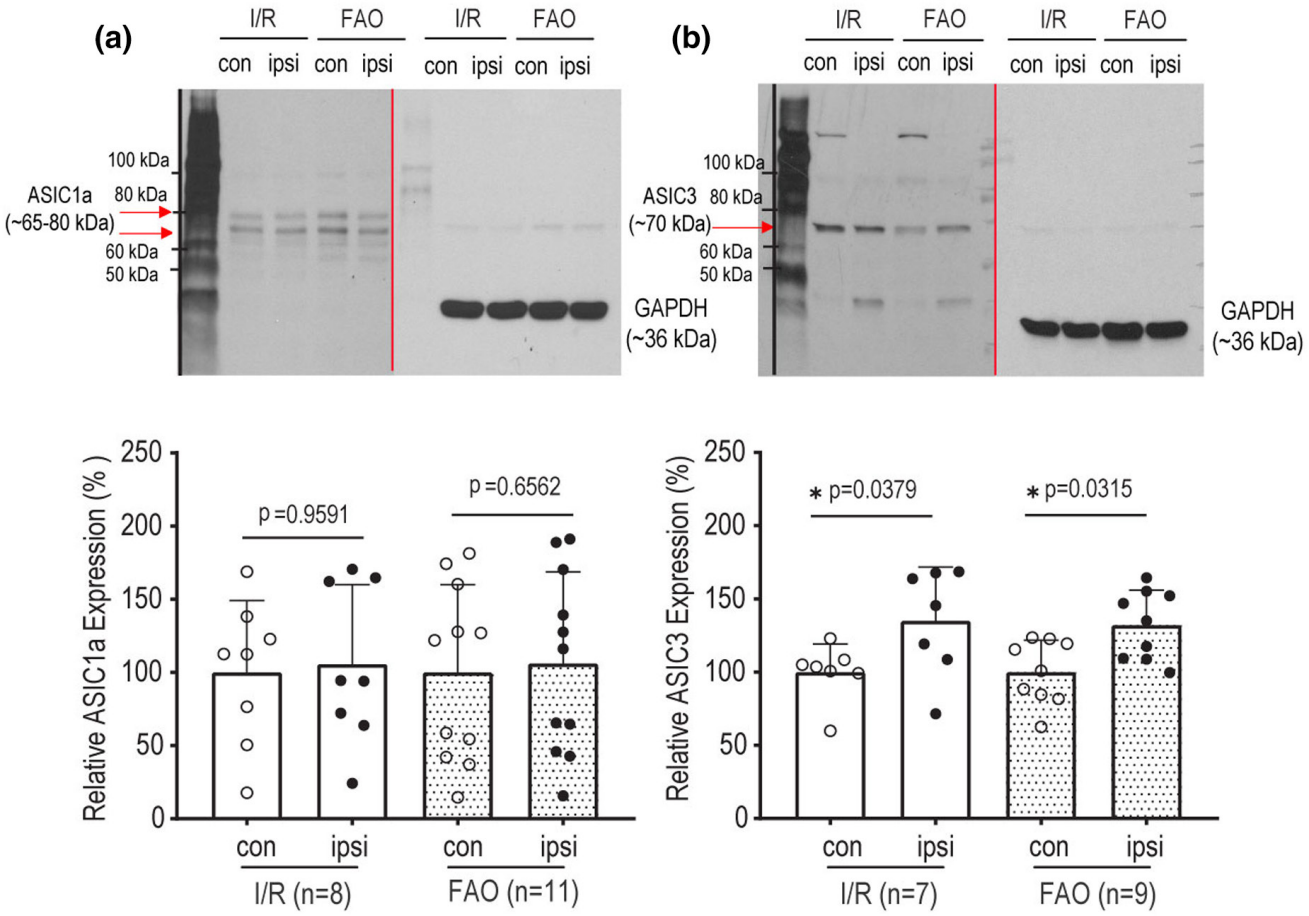
ASICs expression in rat L4‐6 DRGs. (a) ASIC1a expression after ischemia/reperfusion (I/R) and femoral artery occlusion (FAO). The upper panel is the representative bands of ASIC1a from different groups determined by western blotting analysis (vertical lines used to separate the panels of ASIC1a/ASIC3 and GADPH), and the bottom panel is the graph of the relative level of ASIC1a expression comparing to the control. There was no significant difference after I/R and FAO comparing the control group. Un‐paired *t*‐test was used to analyze the data. *p* = 0.9591 between I/R (*n* = 8) and control (*n* = 8); and *p* = 0.6562 between FAO (*n* = 11) and control (*n* = 11). (b) ASIC3 expression in DRGs of I/R and FAO groups. The upper panel illustrates the representative bands of ASIC3, and the bottom panel is the relative level ASIC3 expression comparing to the control. *p < 0.05, comparing between the two groups as indicated. That is, *p* = 0.0379 between I/R (*n* = 7) and its control (*n* = 7); and *p* = 0.0315 between FAO (*n* = 9) and its control (*n* = 9). Both ipsilateral and contralateral sides of the L4‐L6 DRGs from the same rat were removed for I/R and FAO groups. “ipsi” designates the limbs treated with I/R or FAO and “con” designates the contralateral limbs with the sham procedures as respective controls. Individual data are also shown in figures. The membrane was stripped and incubated with mouse anti‐GAPDH primary antibody as an equal load control. Note that the protein was transferred to the PVDF membrane and blocked using anti‐TrkA antibody to detect the protein level of TrkA and stripped using the stripping buffer. Following being blocked using anti‐ASIC3 antibody to detect the protein level of ASIC3, anti‐GAPDH antibody was used for an equal loading control of protein expression and thus the same typical GADPH band was shown in both (b) and Figure 1c as expression of TrkA and ASIC3 was determined by using the experimental samples for western blot. PVDF, polyvinylidene difluoride.

We then performed a novel study to examine the effects of I/R and FAO on expression of ASICs in L4‐6 DRGs. Figure 2a (upper and bottom panels) are the representative ASIC1a bands of western blotting analysis and averaged data, demonstrating that I/R and FAO failed to significantly alter the protein levels ASIC1a expression in L4‐6 DRGs as compared with their respective contralateral controls (*p* = 0.9591, I/R/*n* = 8 vs. its control/*n* = 8); and (*p* = 0.6562, FAO/*n* = 11 vs. its control/*n* = 11). Figure 2b (upper and bottom panels) is the representative ASIC3 bands and averaged data, showing that the protein levels ASIC3 expression were increased in L4‐6 DRGs of I/R limbs and FAO limbs as compared with their respective contralateral control limbs (*p* = 0.0379, I/R/*n* = 7 vs. its control/*n* = 7; and *p* = 0.0315, FAO/*n* = 9 vs. its control/*n* = 9).

### 3.3 Effect of NGF on ASIC currents in muscle DRG neurons

ASIC3 is activated by pH 6.5–7.0 (Deval et al., 2008, 2011), and this range of pH is observed in exercising muscle and/or moderately ischemic tissues (MacLean et al., 1998, 2000; Rotto et al., 1989; Yagi et al., 2006). Thus, in the present study, pH 6.7 was selected to stimulate ASIC3 and ASIC1a, likely involved in the process of pathophysiological responses such as ischemic insult and/or I/R injury as mentioned above.

With application of pH 6.7 solution for 10 s, two distinct types of inward currents were seen in L4‐6 DRGs neurons, termed as ASIC1a‐like and ASIC3‐like currents in this study. Those ASIC currents were distinguished by their inactivation time constant (*τ*_inact_). With standard exponential function, ASIC1a‐like currents had a slower *τ*_inact_ and ASIC3‐like current had a faster *τ*_inact_. In addition, the amplitude of ASIC1a and ASIC3 currents was decreased by PcTx1 (antagonist to ASIC1a channels) and APETx2 (antagonist to ASIC3 channels), respectively (Li et al., 2023). Results of our current study are consistent with the previous reports showing that functional ASIC1a and ASIC3 receptors were present and activated in rat L4‐6 DRG neurons (Farrag et al., 2017; Xing et al., 2012).

**FIGURE 3:**
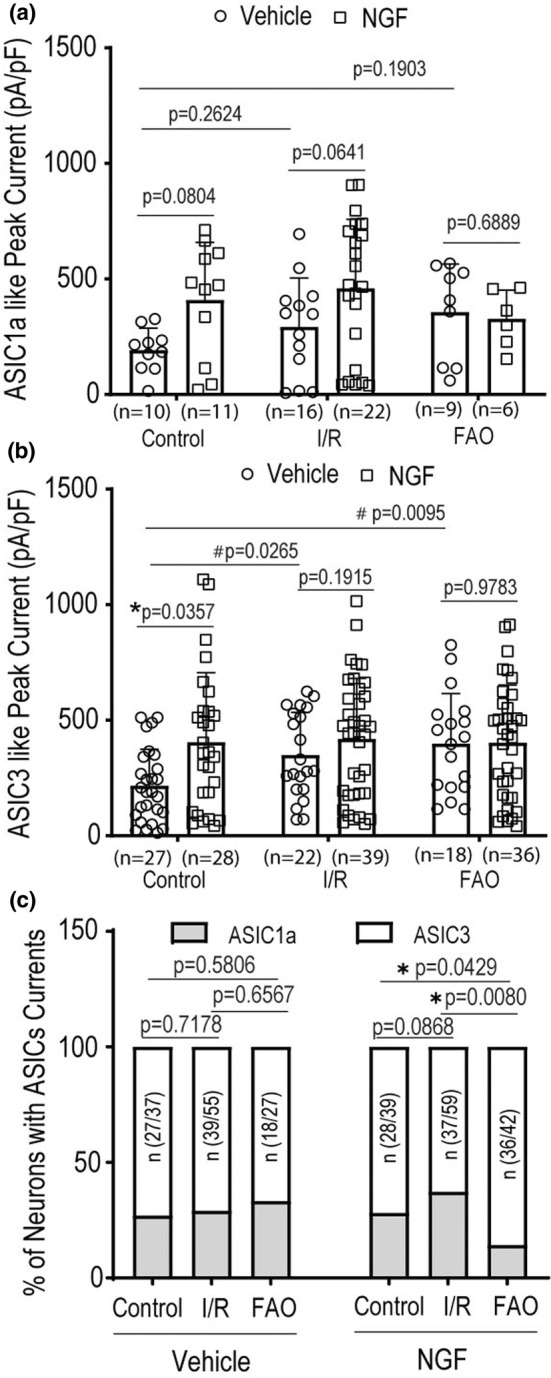
The effect of NGF on the ASICs currents in rat L4‐6 DRG neurons. (a and b) The graphs showing the averaged peak current density of ASIC1a‐like currents and ASIC3‐like currents in rat L4‐6 DRG neurons at pH 6.7. After treatment with 100 ng/mL NGF for 24 h, in each group no significant difference was found in ASIC1a‐like currents comparing to the vehicle, respectively. Two‐way ANOVA with Tukey's tests was used to analyze the data and result indicated no significant main effect (*F* = 3.397 and *p* = 0.070) and interaction (*F* = 1.141 and *p* = 0.3258). *p* = 0.0804 between NGF (*n* = 11) and vehicle (*n* = 10) in control group, *p* = 0.0641 between NGF (*n* = 22) and vehicle (*n* = 16) in I/R group, and *p* = 0.6889 between NGF (*n* = 6) and vehicle (*n* = 9) in FAO group. Control group was obtained from DRG neurons of a different group of rats. However, 100 ng/mL NGF significantly increased ASIC3‐like currents of L4‐6 DRG neurons in control group, but not in IR and FAO groups. Two‐way ANOVA with Tukey's tests was used to analyze the data (*F* = 5.242 and *p* = 0.0233 for main effect; and *F* = 1.708 and *p* = 0.1848 for interaction). *p = 0.0357 between vehicle (*n* = 27) and NGF (*n* = 28). In addition, I/R and FAO significantly amplified the density of ASIC3 currents #*p* < 0.05, I/R and FAO versus control group. *p* = 0.0265 between I/R (*n* = 22) and control (*n* = 27); and *p* = 0.0095 between FAO (*n* = 18) and control (*n* = 27). Individual data and *p*‐values are also shown in figures. Control group was obtained from DRG neurons of a different group of rats. (c) The graphs showing the distribution of ASIC1a‐like currents and ASIC3‐like currents in rat L4‐6 DRG neurons after application of NGF. The distribution of ASIC1a and ASIC3 was analyzed using chi‐squared (*χ* ^*2*^) No significant difference was found in the distribution of ASICs‐like currents after application of the vehicle in the control, IR and FAO groups, respectively. 100 ng/mL NGF significantly increased the proportion of ASIC3‐like currents in FAO group comparing to the control and IR groups. *p < 0.05 between the two groups indicated. The bracketed ratios of *n* represent number of ASIC3 neurons/number of total ASIC neurons (ASIC1a + ASIC3 neurons). *p*‐Values are shown in the figures.

As a result of increases in NGF and expression of NGF's TrkA receptor as well as ASIC3 in DRGs of ischemia limbs, our study further determined if NGF can affect the activities of ASIC currents at pH 6.7 with pre‐application of 100 ng/mL NGF for 24 h (Mamet et al., 2002, 2003). The new data of Figure 3a,b show that the averaged peak current density of ASIC1a‐like currents at pH 6.7 was not significantly altered by NGF in control (*p* = 0.0804 between NGF/*n* = 11 and vehicle control/*n* = 10), I/R (*p* = 0.0641 between NGF/*n* = 22 and vehicle control/*n* = 16), and FAO groups (*p* = 0.6889 between NGF/*n* = 6 and vehicle control/*n* = 9), whereas ASIC3‐like currents in rat L4‐6 DRG neurons at pH 6.7 were increased in control group (*p* = 0.0357 between NGF/*n* = 28 and vehicle control/*n* = 27). Note that the effect of NGF was observed in the control rats, but was not found in the I/R or FAO rats. Nonetheless, I/R and FAO amplified ASIC3‐like currents in L4‐6 DRG neurons as compared with control group (*p* = 0.0265 between I/R/*n* = 22 and control/*n* = 27; and *p* = 0.0095 between FAO/*n* = 18 and control/*n* = 27 as shown in Figure 3b).

Figure 3c demonstrates the distribution of ASIC1a‐like currents and ASIC3‐like currents in rat L4‐6 DRG neurons. No significant difference was found in the distribution of ASICs‐like currents caused by vehicle in the control, IR, and FAO groups (*p* > 0.05 among all the groups). However, 100 ng/mL NGF significantly increased the proportion of ASIC3‐like currents in FAO group comparing to the control and IR groups (*p =* 0.0429, FAO vs. control; and *p =* 0.0080, FAO vs. I/R).

### 3.4 The role of TrkA for NGF regulating ASIC currents

**FIGURE 4:**
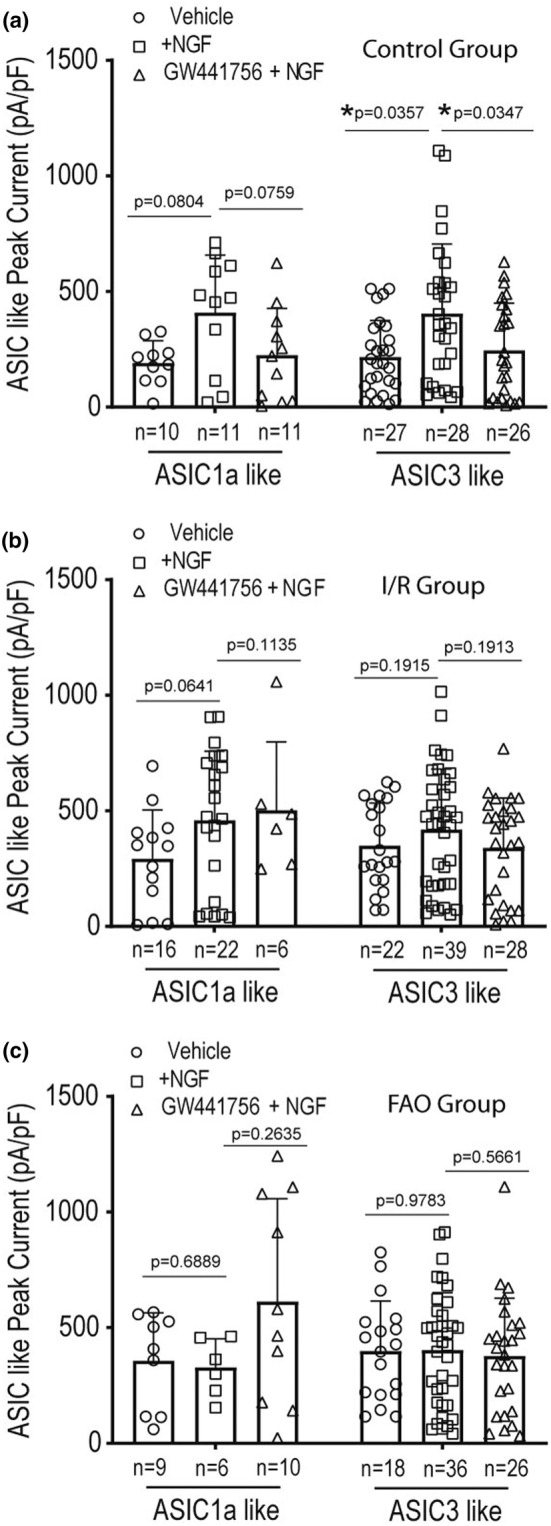
NGF‐affected ASICs currents via TrkA. The effect of NGF on ASICs currents was examined after GW441756, an inhibitor of TrkA, was applied onto rat L4‐6 DRG neurons for 4 h before 100 ng/mL NGF treatment. (a) The graph of the averaged peak density of ASICs‐like currents in rat L4‐6 DRG neurons at pH 6.7 in the control group. Control group was obtained from DRG neurons of a different group of rats. The pretreatment of 1 μM GW441756 significantly attenuated the amplified ASIC3‐like currents induced by 100 ng/mL NGF, but this did not significantly affect ASIC1a‐like currents. Two‐way ANOVA with Tukey's tests was used to analyze the data and results indicated significant main effect (*F* = 7.583 and *p* = 0.0008) and but not interaction (*F* = 0.03797 and *p* = 0.9628). *p < 0.05 between the two groups indicated. *p* = 0.0347 between NGF (*n* = 28) and NGF + GW441756 (*n* = 26); and *p* = 0.0357 between NGF (*n* = 28) and vehicle (*n* = 27). (b and c) The graphs show the averaged peak density of ASICs‐like currents in rat L4‐6 DRG neurons at pH 6.7 in IR group and in FAO group. There was no significant difference in density of ASIC1a‐like currents and ASIC3‐like currents between group with pretreatment of 1 μM GW441756 plus 100 ng/mL NGF and group with 100 ng/mL NGF alone. Two‐way ANOVA with Tukey's tests was used to analyze the data (*F* = 2.444 and *p* = 0.0910 for main effect; *F* = 1.225 and *p* = 0.2973 for interaction in I/R group; and *F* = 1.974 and *p* = 0.1444 for main effect and *F* = 2.803 and *p* = 0.0654 for interaction in FAO group). Individual data, sample size and *p* values between groups indicated in control, I/R and FAO are shown in the figures.

The effect of NGF on ASICs currents in rat L4‐6 DRG neurons was also examined after blocking NGF‐activated TrkA receptors in control, I/R, and FAO groups. The new findings are presented in Figure 4. That is, Figure 4a shows that the pretreatment of 1 μM GW441756, an inhibitor of TrkA for 4 h (Baldassarro et al., 2023; Terada et al., 2018), significantly decreased amplification of ASIC3‐like currents induced by pre‐application of 100 ng/mL NGF for 24 h in the control group (*p* = 0.0347 between GW441756 + NGF/*n* = 26 and NGF/*n* = 28). Note that the pretreatment of 1 μM GW441756 did not alter ASIC1a current density in L4‐6 DRG neurons of all the groups. Figure 4b,c further demonstrate that 1 μM GW441756 did not significantly alter the averaged density of ASIC1a‐like currents and ASIC3‐like currents in rat L4‐6 DRG neurons of IR and FAO groups.

**FIGURE 5:**
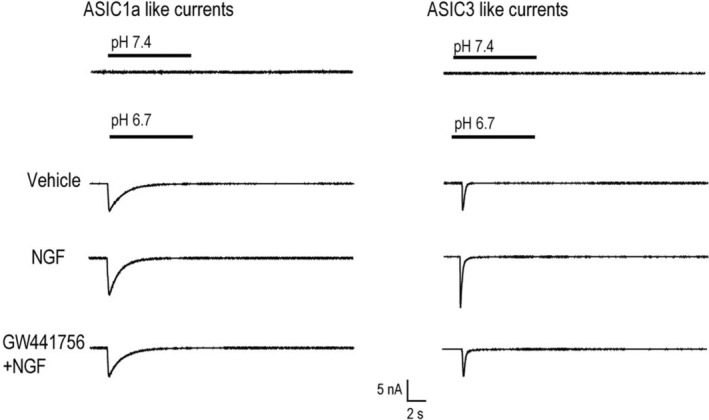
The representative traces of ASIC1a‐like currents and ASIC3‐like currents in L4‐6 DRG neurons. Each trace was recorded from one individual L4‐6 DRG neuron of control rats after vehicle, 100 ng/mL NGF and NGF with pre‐application of 1 μM GW441756, respectively. ASICs were activated with pH 6.7 and pH 7.4 was applied for controls.

The representative traces of ASIC1a‐ and ASIC3‐like currents are illustrated in Figure 5, indicating the enhancing effects of NGF on the amplitude of ASIC3 currents in L4‐6 DRG neurons of control rats.

### 3.5 Intracellular signaling pathways responsible for the effect of NGF on ASIC currents

**FIGURE 6:**
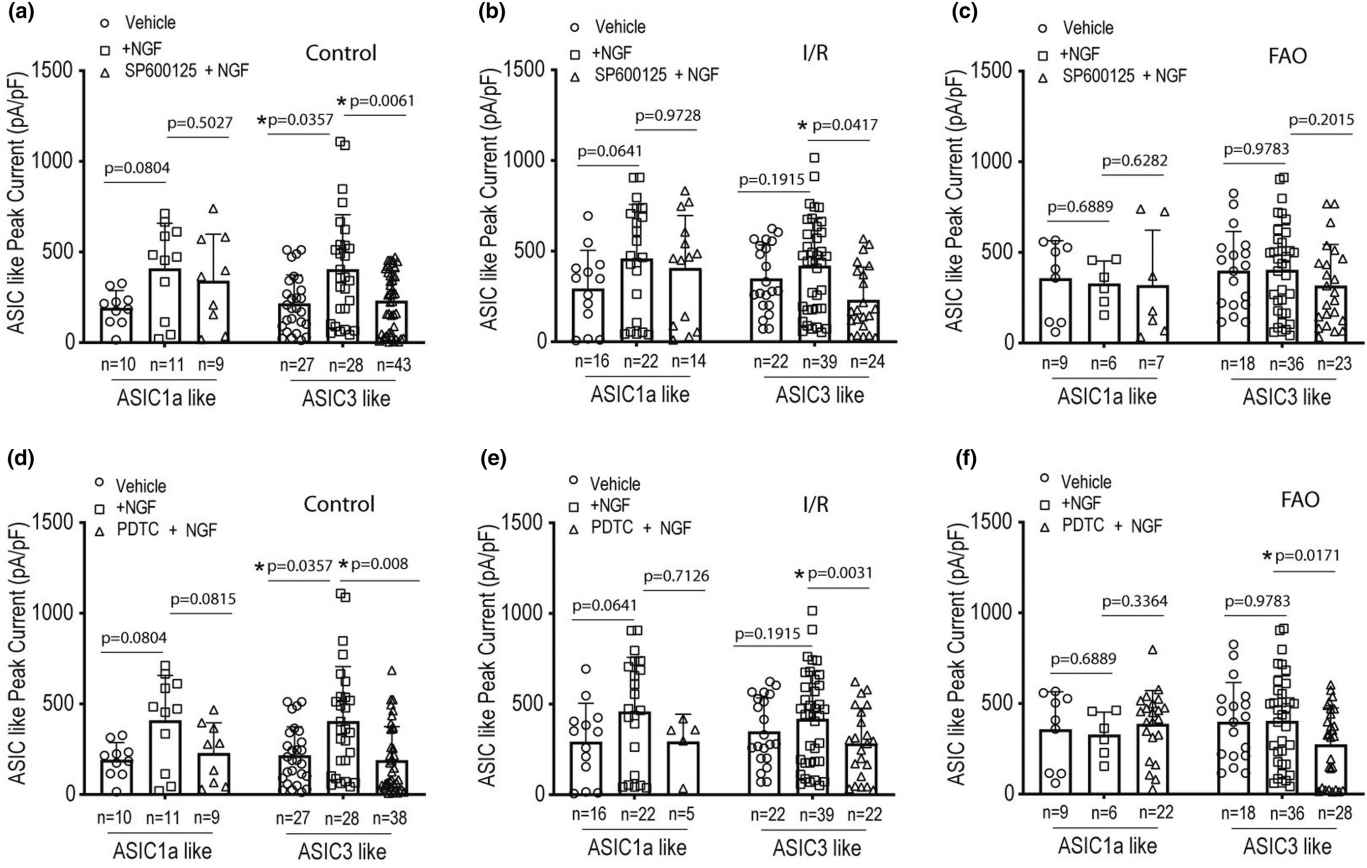
Intracellular signaling pathways for the effect of NGF on ASIC currents. (a–c) A JNK inhibitor, SP600125, was applied onto L4‐6 DRG neurons for 4 h before determining the effect of NGF on ASICs currents in control, I/R, and FAO groups. The graphs of the averaged peak density of ASICs‐like currents in rat L4‐6 DRG neurons at pH 6.7, showing that the pretreatment of 10 μM SP600125 significantly decreased the amplified ASIC3‐like currents by 100 ng/mL NGF in the control rat DRG neurons (*p = 0.0061 between NGF and NGF + SP600125; two‐way ANOVA: *F* = 7.713 and *p* = 0.0012 for main effect; and *F* = 0.8396 and *p* = 0.4344 for interaction), but this did not affect ASIC1a‐like currents (a). A significant attenuation on ASIC3‐like currents was also found in IR group after application SP600125 compared with NGF alone (b). *p = 0.0417 between the two groups as indicated (two‐way ANOVA: *F* = 3.598 and *p* = 0.0303 for main effect; and *F* = 1.902 and *p* = 0.1537 for interaction). No significant difference in ASIC3‐like currents was found in FAO group after application SP600125 compared with NGF alone. Two‐way ANOVA indicated: *F* = 0.4573 and *p* = 0.6341 for main effect; and *F* = 0.1490 and *p* = 0.8617 for interaction. *p* = 0.2015 between the two groups as indicated (c). All the *p* values are shown in the figures. Control group was obtained from DRG neurons of a separate group of rats. The effect of the NF‐κB inhibitor, PDTC, on NGF‐activated ASICs currents in rat L4‐6 DRG neurons was also examined in control, I/R, and FAO groups (d–f) The graphs of the averaged peak density of ASICs‐like currents in rat neurons at pH 6.7, showing that the pretreatment of 10 nM PDTC for 4 h significantly attenuated the increased ASIC3‐like currents induced by 100 ng/mL NGF in the control rat DRG neurons, but did not affect ASIC1a‐like currents. Moreover, there was a significant difference in ASIC3‐like currents observed as compared between the pretreatment of PDTC and NGF alone in IR group and FAO group. The pretreatment of PDTC was not found to significantly affect ASIC1a‐like currents in IR and FAO group. Two‐way ANOVA was used to analyze the data. *F* = 9.150 and *p* = 0.0002 (main effect); and *F* = 0.1725 and *p* = 0.8418 (interaction) in control group. *F* = 3.907 and *p* = 0.0228 (main effect); and *F* = 0.4141 and *p* = 0.6620 (interaction) in I/R group. *F* = 3.486 and *p* = 0.0355 (main effect) and *F* = 0.9110 and *p* = 0.4063 (interaction) in FAO group. All the *p* values are presented in the figures and * is indicated *p* < 0.05 between the two groups as indicated. Control group was obtained from DRG neurons of a separate group of rats.

Moreover, in this study we determined intracellular signaling pathways, namely JNK, in involvement of the effects of NGF on the activities of ASIC currents. The novel data of Figure 6a,b demonstrate that the pretreatment of 10 μM SP600125 (JNK inhibitor) for 4 h (Li et al., 2020; Lirk et al., 2008), significantly attenuated the increased ASIC3‐like currents evoked by NGF treatment in L4‐6 DRG neurons of control (*p* = 0.0061 between NGF + SP600125/*n* = 43 and NGF alone/*n* = 28) and in L4‐6 DRG neurons of I/R group (*p* = 0.0417 between NGF + SP600125/*n* = 24 and NGF alone/*n* = 37). The same pretreatment of SP600125 failed to alter ASIC1a‐like currents in L4‐6 DRG neurons of control, I/R, and FAO groups (Figure 6a–c).

NGF has a regulatory effect on ASIC currents in sensory neurons via NF‐κB signaling pathway (Wei et al., 2020; Xie et al., 2019). Our new findings further demonstrate that the effect of pyrrolidinedithiocarbamate ammonium (PDTC, NF‐κB inhibitor) on NGF‐activated ASICs currents in rat L4‐6 DRG neurons. The averaged data for peak density of ASICs‐like currents in rat L4‐6 DRG neurons at pH 6.7 (Figure 6d–f) show that the pretreatment of 10 nM of PDTC for 4 h significantly decreased density of ASIC3‐like currents induced by NGF treatment in L4‐6 DRG neurons of the control, I/R, and FAO groups (*p =* 0.008, PDTC+NGF/*n* = 38 vs. NGF alone/*n* = 28 in control group; *p =* 0.0031, PDTC+NGF/*n* = 22 vs. NGF alone/*n* = 37 in I/R group; and *p =* 0.0171, PDTC+NGF/*n* = 28 vs. NGF alone/*n* = 36 in FAO group). However, the pretreatment of 10 nM of PDTC did not alter ASIC1a‐like currents in those groups (Figure 6d–f).

## 4 DISCUSSION

ASICs currents are largely found in rodent lumbar DRG neurons (Dirajlal et al., 2003; Poirot et al., 2006) and ASICs currents mostly appear in IB4− neuronal cells (Averill et al., 1995; Bennett et al., 1998; Michael et al., 1997; Molliver et al., 1997). Note that the hindlimb I/R and FAO in rats affect ASIC signaling pathway in IB4− muscle DRG neurons to a greater degree than that in IB4+ muscle DRG neurons (Li et al., 2023). Our data also suggest that ASIC3‐like currents are predominant as compared with ASIC1a‐like currents in L4‐6 DRG neurons in contribution to the activities of ASICs with pH 6.7. Thus, in the current study it is interesting to determine if NGF has a different effect on ASIC3 versus ASIC1a in IB4− L4‐6 DRG neurons of hindlimb muscle with I/R and FAO.

In FAO rats, ASIC3 in L4‐6 DRGs was seen and this was consistent with the increases of ASIC3 current activities in the DRG neurons (Farrag et al., 2017; Liu et al., 2010; Xing et al., 2012). A study using ASIC3 KO rats also suggests the role of ASIC3 in the exaggerated EPR in PAD (Kim et al., 2020). ASIC1a plays a role in the EPR in normal rats (Ducrocq et al., 2020a, 2020b). Notably, a human study further suggests that ASICs are engaged in the EPR (Campos et al., 2019). Our first data of the present study demonstrated that FAO increased NGF (Figure 1a) and its receptor TrkA in DRGs (Figure 1c) as well as protein expression of ASIC3 but not ASIC1a (Figures 2a,b), suggesting that NGF is likely to play a role in regulating the activities of ASIC3 in the DRG neurons of experimental PAD rats. Nonetheless, the subsequent question was how NGF affected the activities of ASIC currents in the DRG neurons of PAD. We then examined the effects of NGF on ASIC1a and ASIC3 currents in IB4− L4‐6 DRG neurons (Figures 3) as both ASIC1a and ASIC3 receptors largely appear in the sensory nerves and play a functional role in regulating neural signaling conduction.

In our previous study, our data showed how ASICs regulate the EPR in PAD (Liu et al., 2010). Using respective antagonists to ASIC1a and ASIC3, we also characterized ASIC1a‐like currents and ASIC3‐like currents recorded in rat L4‐6 DRG neurons, and PcTx1 and APETx2 attenuated ASIC1a and ASIC3 currents, respectively (Li et al., 2023). The distinguished inactivation time constant was used to examine ASIC1a and ASIC3 currents with activation of pH 6.7. Consistently, in the current study we observed ASIC1a‐like currents with a slower inactivation time constant and ASIC3‐like current with a faster time constant when pH 6.7 was applied onto L4‐6 DRG neurons. Moreover, NGF increased the density of ASIC3 currents but not ASIC1a (Figures 3a,b). This result was observed in L4‐6 DRG neurons of control limbs but not in DRG neurons of I/R and FAO limbs. In addition, pre‐application of TrkA antagonist GW441756 onto L4‐6 DRG neurons significantly attenuated amplification of ASIC3 currents induced by NGF (Figure 4a), indicating that NGF‐TrkA pathways are involved in the effects of NGF on the activities of ASIC3 in the DRG neurons. A less effect of NGF on the activities of ASIC3 current in L4‐6 DRG neurons of I/R and FAO limbs (Figure 3b) was likely due to overexpression of TrkA and ASIC3 receptors which desensitize NGF stimulation when it was applied onto the neurons (Sherwood et al., 2012). In another word, protein expression of ASIC3 may not totally reflect its activities or sensitivities in L4‐6 DRG neurons.

It is interesting that NGF increased the population of ASIC3‐like currents in L4‐6 DRG neurons of FAO rats (Figure 3c) and this was accompanied with increased ASIC3 protein expression (Figure 2b). However, one may have a question why no changes were observed in ASIC1a expression (Figure 2a) even though a relative decrease in the population of ASIC1a currents (Figure 3c). It is assumed that a decrease in the population of ASIC1a (determined by its current activity) is not sufficient to alter protein expression of ASIC1a. On the other hand, it could be explained by which protein expression of a certain channel does not reflect its activities or sensitivities. Regarding the effect of NGF on % distribution of ASIC currents, intracellular pathways such as JNK and NF‐κB might be involved to play a role.

In the current study, our data further showed that JNK inhibitor SP600125 and NF‐κB inhibitor PDTC attenuated ASIC3 currents in L4‐6 DRG neurons of control, I/R, and FAO groups after NGF application (Figures 6a–f). This supports the general idea that NGF play a role in regulating the activities of ASIC3 currents via JNK and NF‐κB signaling pathways (Mamet et al., 2003; Matricon et al., 2013; Wei et al., 2020). This mechanism is likely involved in the exaggerated EPR in PAD.

Nonetheless, blocking ASIC1a can alleviate the BP response to muscle contraction in normal rats, but this was not seen in rats with FAO (Ducrocq et al., 2020a, 2020b). Evidence cannot be conclusive for the role of ASIC1a in regulating the EPR in PAD; however, based on the previous findings we think that there is a discrepancy in the activities of ASIC1a and ASIC3 in IB4− versus IB4+ DRG neurons, particularly their activation with a low pH following I/R and FAO (Li et al., 2023). In a previous study, in mice brachial artery I/R was observed to amplify mRNA ASIC1a and ASIC3 in cervical and C7/C8/T1 DRGs innervating the forelimb muscle (Ross et al., 2014). BP response during dynamic exercise was also exaggerated in I/R mice (Queme et al., 2020). In contrast, in the decerebrated rats with IR, the exaggerated EPR was also seen during static muscle contractions (Qin & Li, 2022). According to those previous findings, it is interesting to study ASICs currents in L4‐6 DRG neurons under the I/R and FAO conditions of the PAD rats. We found that the activities of ASIC3‐like current in rat IB4− DRG neurons after the hindlimb I/R and FAO were significantly amplified. A distribution of ASIC1a currents was greater in IB4+ ASICs neurons of I/R rat at pH 5.5, but not in IB4− ASICs neurons (Li et al., 2023). Overall, those accumulated data are helpful for elucidation of the roles played by ASIC1a and ASIC3 in modulating the EPR in I/R injury and ischemia seen in PAD and suggest that the levels of muscle pH should be considered for explanation of the experimental findings.

A study limitation needs to be known that currently reported sample size may be contributing to the lack of significant findings involving the ASIC1a currents. Specifically, the distinct effects of NGF on ASIC1a currents (i.e., Figures 3a, 4, and 6) may be seen with increasing the number of DGR neurons.

## 5 CONCLUSION

It was observed that the protein levels of both TrkA and ASIC3 receptors were increased in L4‐6 DRGs following the limb I/R and ischemia in PAD rats. Via NGF‐TrkA signaling pathways the activities of ASIC3 are amplified in IB4− DRG neurons. In the process of NGF signaling, intracellular JNK and NF‐κB are found to be engaged in the effects of NGF on the activities of ASIC3 under the conditions of limb I/R injury and ischemia of PAD. Overall, the role played by NGF in regulating ASICs should be considered to study the exaggerated EPR in PAD.

## AUTHOR CONTRIBUTIONS

Q. Li contributed to data collection and analysis of experimental data and drafted the manuscript. J. Li designed study, oversaw performance of the experiments and data analysis, and drafted and revised the manuscript. Both authors approved the final version of the manuscript submitted for publication.

## FUNDING INFORMATION

This study was supported by NIH R01 HL141198 & HL164571 (to Jianhua Li), and Penn State College of Medicine DOM Innovation and Inspiration Award (INNOVQLI Fall2021 to Qin Li).

## CONFLICT OF INTEREST STATEMENT

The authors declare no conflict of interest.

## ETHICS STATEMENT

All experimental procedures were approved by the Institutional Animal Care and Use Committee of Penn State College of Medicine (Protocol#: PRAMS201147671) and were conducted in accordance with the National Institutes of Health Guide for the Care and Use of Laboratory Animals.

## Data Availability

Appropriate data will be provided by individual requests in accordance with the grant funding agency.
